# MiR-216b is involved in pathogenesis and progression of hepatocellular carcinoma through HBx-miR-216b-IGF2BP2 signaling pathway

**DOI:** 10.1038/cddis.2015.46

**Published:** 2015-03-05

**Authors:** F-y Liu, S-j Zhou, Y-l Deng, Z-y Zhang, E-l Zhang, Z-b Wu, Z-y Huang, X-p Chen

**Affiliations:** 1 Department of Surgery, Wuhan Center Hospital, Wuhan, Hubei, China; 2 Research Laboratory and Hepatic Surgical Center, Department of Surgery, Tongji Hospital, Tongji Medical College, Huazhong University of Science and Technology, Wuhan, HuBei, China; 3 Department of Gastroenterology, Wuhan Center Hospital, Wuhan, Hubei, China

## Abstract

This study aims to investigate the expression status of miRNA-216b in familial hepatocellular carcinoma (HCC) and the correlation between miRNA-216b expression and pathogenesis, as well as the progression of HCC. The expression profile of miRNAs in plasma of peripheral blood between HCC patients with HCC family history and healthy volunteers without HCC family history was determined by microarray. Using real-time quantitative PCR to detect the expression in paired tissues from 150 patients with HCC, miR-216b was selected as its expression value in HCC patients was significantly lower compared with healthy volunteers. Next, miR-216b expression and the clinicopathological features of HCC were evaluated. The effect of miR-216b expression on tumor cells was investigated by regulating miR-216b expression in SMMC-7721 and HepG2 *in vitro* and *in vivo*. Finally, we explored mRNA targets of miR-216b. In 150 HCC, 37 (75%) tumors showed reduced miR-216b expression comparing with their adjacent liver tissues. The decreased expression of miR-216b was significantly correlated with tumor volume (*P*=0.044), HBV infection (*P*=0.026), HBV DNA quantitative (*P*=0.001) and vascular invasion (*P*=0.032). The 5-year disease-free survival and overall rates after liver resection in low expression and high expression groups of miR-216b are 62% and 54%, 25% and 20%, respectively. MiR-216b overexpression inhibited cell proliferation, migration and invasion, and miR-216b inhibition did the opposite. The expression of hepatitis B virus x protein (HBx) has tight correlation with downregulation of miR-216b. Furthermore, miR-216b downregulated the expression of insulin-like growth factor 2 mRNA-binding protein 2 (IGF2BP2) and exerted its tumor-suppressor function through inhibition of protein kinase B and extracellular signal-regulated kinase signaling downstream of IGF2. MiR-216b inhibits cell proliferation, migration and invasion of HCC by regulating IGF2BP2 and it is regulated by HBx.

Hepatocellular carcinoma (HCC) is one of the most common malignancies and is the third most common cause of cancer-related deaths worldwide.^1^ China accounts for >50% of the total incidence of HCC in the world.^2^ Most patients with HCC are diagnosed at an advanced stage that renders surgical therapy ineffective. Prognosis of HCC is poor even among patients who undergo liver resection, with 5-year cumulative tumor recurrence rate being 77–100%.^3^ Chronic hepatitis B virus (HBV) infection accounts for approximately 50% of the total cases of adult HCC and almost all cases of childhood HCC.^4^ Several studies have suggested that inherited factors influence the risk of developing HCC. Multivariate adjusted hazard ratio for the comparison of hepatitis B virus surface antigen (HBsAg)-seropositive individuals with family history of HCC and HBsAg-seronegative individuals without a family history of HCC is 32.33.^5^ Demir *et al.*^6^ reported a case identical twin brothers who were diagnosed with HCC at the same time and who were unresponsive to chemotherapy and died within the same month. Another study showed that the probability of HBV-associated HCC to be resectable is influenced by the family history of HCC. Particularly, if a patient's sibling has a history of HBV infection, the patient is more likely to develop unresectable HCC.^7^ However, the mechanism underlying this association is unknown.

MicroRNAs (miRNAs) are non-coding RNAs that interact directly with the 3′-untranslated region (3′-UTR) of target mRNAs ^8, 9^ and inhibit gene expression by inhibiting the translation of these target mRNAs or by degrading them.^10^ MiRNAs perform pleiotropic functions by affecting proliferation, differentiation, metastasis and apoptosis. Studies have suggested that altered miRNA expression is associated with cancer.^11, 12^ MiRNAs may act as oncogenes or tumor suppressors; their functions vary depending on the organs and tumors in which they are expressed.^13^ MiRNA expression in the plasma or tumor cells of patients with HCC and healthy controls is commonly measured to screen novel miRNAs associated with the pathogenesis and progression of HCC. Our study differs from this strategy, in that we examined miRNA expression in the plasma of patients with HCC who had a family history of HBV-associated HCC and healthy volunteers and identified miRNAs with significantly altered expression levels. Further, we validated these miRNAs by measuring their expression in tumors tissues and adjacent liver tissues. Finally, we determined the molecular functions of these miRNAs and identified their underlying mechanisms by using HCC cell lines, nude mice and patients with HCC.

## Results

### MiRNA profiles of the plasma of patients with HCC who had a family history of HBV-associated HCC

**Figure 1:**
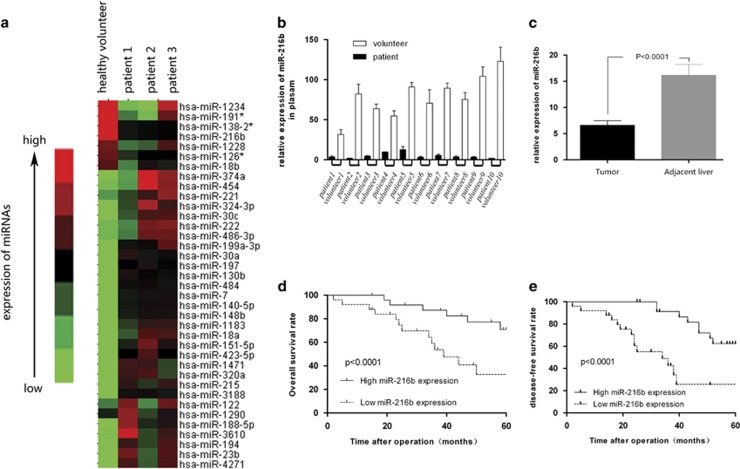
MiR-216b is downregulated in HCC tissues and plasma, direct correlation with prognosis of HCC patients. (**a**) miRNA array test of the expression levels of all kinds of miRNAs in plasma of HCC patients with HCC family history. (**b**) RT-PCR analysis of the expression levels of miR-216b between 10 pairs of HCC patients and healthy volunteer's plasma. The miR-216b expression levels in healthy volunteer plasma were much higher than in HCC patients' plasma. (**c**) RT-PCR analysis of the expression levels of miR-216b in 150 pairs of HCC patients' tissues and contrast the expression value between tumor and adjacent liver tissues. The expression of miR-216b in tumor tissues was significant higher than in adjacent liver. (**d** and **e**) After operation, the 5-year cumulative survival rate and disease-free survival rate of low miR-216b expression group is much lower than high expression group, and *P*<0.05

Agilent MiRNA Base 16.0 microarray showed 45 significant differential expression miRNAs between HCC patients and healthy volunteers, of which 26 miRNAs showed >20-fold difference in expression (Table 1). Of these 26 miRNAs, 12 have been reported previously in HCC or other tumors (Supplementary Table 2). Of the remaining 14 miRNAs, miR-216b showed 127.56-fold decreased expression in the plasma of patients with HCC compared with that of healthy controls (Supplementary Table 3 and Figure 1a). This result was validated by measuring miR-216b expression in 10 patients with HCC and 10 healthy volunteers by using real-time PCR (Figure 1b). In addition, miR-216b expression was measured in 150 HCC tissues and adjacent liver tissues. We found that miR-216b expression was significantly lower in tumor tissues than in adjacent liver tissues (*P*<0.05; Figure 1c).

### MiR-216b expression is correlated with the biological features and prognosis of HCC

Tumor size, HBV infection, HBV DNA quantity and incidence of portal vein tumor thrombosis (PVTT) were significantly correlated with the low miR-216b expression (Table 2). Five-year overall survival rate was 62% in the high miR-216b expression group and 25% in the low miR-216b expression group (*P*<0.001; Figure 1d). Five-year disease-free survival rate was 54% in the high expression group and 20% in the low expression group (*P*<0.001; Figure 1e).

Univariate analyses were used to analyze miR-216b expression and other clinicopathological parameters on the prognosis of patients with HCC. Multivariate analyses showed that low miR-216b expression (*P*=0.049) and PVTT (*P*=0.008) were independent risk factors of poor prognosis of patients with HCC (Table 3). This suggested that miR-216b expression level could be used as a prognostic indicator in patients with HCC.

### MiR-216b suppresses cell proliferation both *in vitro* and *in vivo*

**Figure 2:**
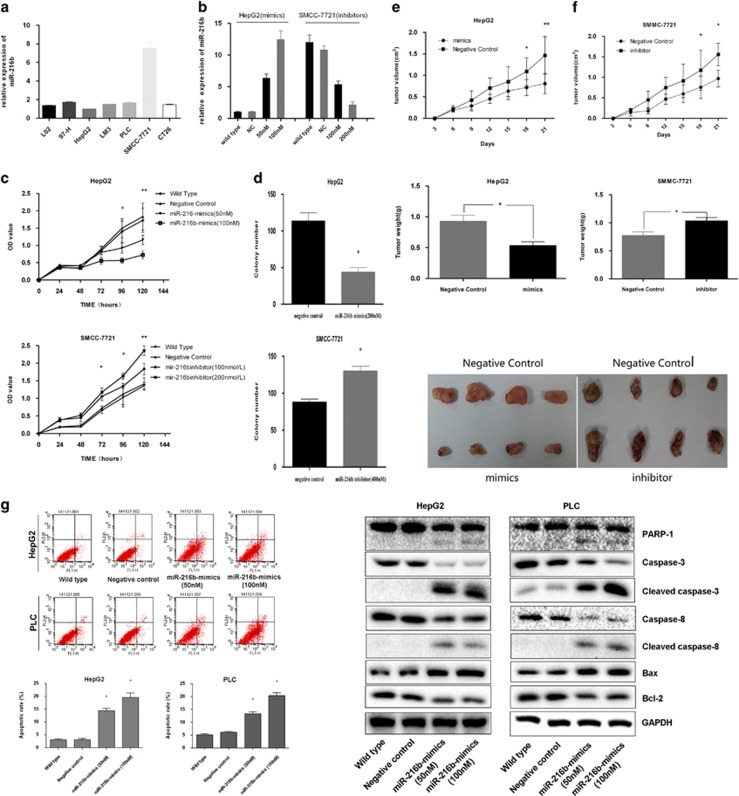
MiR-216b suppresses cell proliferation both *in vitro* and *in vivo*. (**a**) MiR-216b expression is lowest in HepG2 cells and highest in SMCC-7721 cells. (**b**) The mimics and inhibitors of miR-216b could significantly affect the expression of miR-216b and the negative control RNAs (NC) had no obvious effect on expression of miR-216b. (**c**) By CCK-8 assay, the miR-216b upregulated HepG2 cells have smaller value than control cells and Wild Type (WT) cells, and miR-216b downregulated SMCC-7721 cells have larger value than control cells and WT cells. (**d**) Soft agar colony formation assay shows the suppression role of miR-216b in HCC cells' proliferation. (**e** and **f**) MiR-216b attenuated HCC tumor growth in mouse xenograft models. The left panels show tumor formation upon subcutaneous injection of HepG2 cells that were continually transfected with the miR-216b mimics or control vector into nude mice. The right panels show tumor formation upon transplantation of SMCC-7721 cells was continually transfected with the miR-216b inhibitor or control vector into nude mice. (**g**) Flow cytometry study indicating the apoptotic rates in miR-216b overexpressing cells and control cells. MiR-216b induced apoptosis by regulating the important factors of the extrinsic apoptotic pathway

Of the investigated cell lines, HepG2 showed the lowest miR-216b expression and SMMC-7721 showed the highest miR-216b expression (Figure 2a). Therefore, these two cell lines were chosen for further investigation. First, we explored the effect of forced miR-216b expression in HepG2 cells. After transfection, level of miR-216b expression was measured (Figure 2b). After 120 h, the number of cells in the transfected group was significantly lower than that in the control group (*P*<0.05; Figure 2c, top panel). Next, we explored the effect of miR-26b inhibition on SMMC-7721 cells. CCK-8 assay was performed to assess any change in the proliferation of SMMC-7721 cells transfected with miR-216b inhibitors (Figure 2b). After 120 h, the number of cells in the transfected group was significantly higher than that in the control group (*P*<0.05; Figure 2c, bottom panel). Soft agar assay was performed to explore the effects of miR-216 expression on cell colony formation. After 14 days, the number of colonies of HepG2 cells transfected with miR-216b mimics was significantly lower than the number of colonies of control cells. In contrast, the number of colonies of SMMC-7721 cells transfected with 216b inhibitors was significantly higher than the number of colonies of control cells (Figure 2d).

Functions of miR-216b were confirmed using nude mice xenograft models. Nude mice aged 2 weeks were divided into four groups, namely, HepG2 mimics, HepG2 control, SMMC-7721 inhibitors and SMMC-7721 control, with five mice in each group. Mice in first two groups received HepG2 cells transfected with miR-216b mimics and negative control, respectively, whereas those in the remaining two groups received SMMC-7721 cells transfected with miR-216b inhibitors and negative control, respectively. After 21 days, all the tumors were removed and assessed. As shown in Figures 2e and f, mean volumes of resected tumors obtained from mice receiving HepG2 (1.49±0.39 cm3) and SMMC-7721 negative controls (0.93±0.14 cm3) were significantly different from those obtained from mice receiving HepG2 cells transfected with miR-216b mimics (0.75±0.11 cm3) and SMMC-7721 cells transfected with miR-216b inhibitors (1.54±0.41 cm3; *P*<0.01). In addition, a significant difference was observed in tumor weights (*P*<0.05), which was consistent with the above findings. Flow cytometry was performed to investigate whether apoptosis was also involved in the miR-216b-induced inhibition of cell growth. Our data indicated that apoptotic rates of HepG2, HepG2-NC, HepG2-miR-216b mimics (50 nM) and HepG2-miR-216b mimics (100 nM) were 3.05±0.21%, 3.09±0.29%, 14.49±0.53% and 19.6±0.99%, respectively, whereas those of PLC, PLC-NC, PLC-miR-216b mimics (50 nM) and PLC-miR-216b mimics (100 nM) were 5.17±0.35%, 6.13±0.35%, 13.32±0.67% and 20.33± 1.2%, respectively. Significant difference in cell apoptotic rate was observed between wild-type or vector-transfected cells and miR-216b mimics-transfected cells (*P*<0.05). Western blotting showed that miR-216b induced apoptosis through an extrinsic apoptotic pathway (Figure 2g).

### MiR-216b suppresses HCC cell proliferation and invasion by targeting IGF2BP2

**Figure 3:**
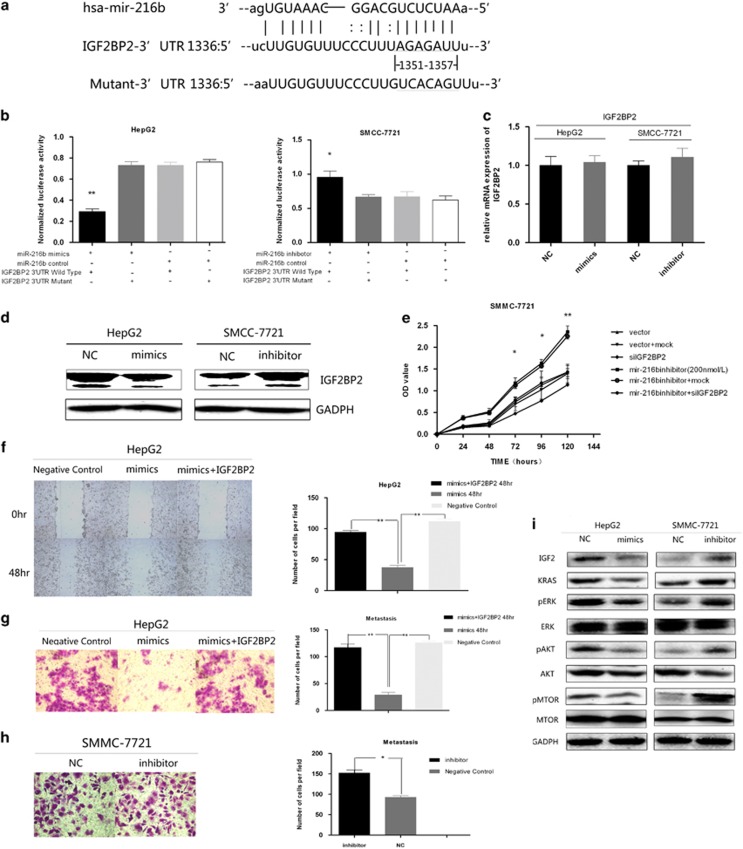
IGF2BP2 is a direct target of miR-216b. (**a**) Sequence alignment of miRNAs of the miR-216b with the IGF2BP2 3'-UTR. The seed-recognizing sites in the IGF2BP2 15 matched bases with the seed regions of miR-216b. (**b**) Luciferase assay on HepG2 cells and SMCC-7721 cells show that miR-216b mimics markedly suppressed luciferase activity in wild-type reporter constructs. MiR-216b inhibitor markedly promoted luciferase activity in wild-type constructs. The data are means ±S.D. (**c**) By RT-PCR, the level of IGF2BP2 mRNA was not affected by miR-216b or anti-miR-216b transfection. All data are shown as means ±S.D. (**d**) MiR-216b or anti-miR-216b transfection affects IGF2BP2 protein levels and the expression of IGF2BP2 was negative correlated with the expression of miR-216b. HepG2 cells were transfected with miR-216b mimics or negative control (NC), and SMCC-7721 cells were transfected with miR-216b inhibitor or NC. (**e**) The CCK-8 assay further showed that the knockdown of IGF2BP2 by si-IGF2BP2 overcame the growth promotion induced by miR-216b inhibitor in SMCC-7721 cells after days 5 or 4 of culture. (**f**) After 48 h, overexpression of miR-216b suppressed HepG2 cell motility by wound-healing assay, but the overexpression of IGF2BP2 could offset this effect. (**g**) Overexpression of miR-216b suppressed HepG2 cell invasion by transwell assay, but the overexpression of IGF2BP2 could offset this effect. (**h**) miR-216b knockdown increased cell invasion in SMCC-7721 cells. All cells were subjected to a Matrigel invasion assay. (**i**) MiR-216b overexpression reduced the activity of AKT/mTOR and MAPK/ERK pathways in HepG2 cells. Knockdown of miR-216b by inhibitor increased the activity of AKT/mTOR and MAPK/ERK signaling in SMCC-7721 cells. All data are shown as means ±S.D. *P<0.05; **P<0.01

To study the mechanism by which miR-216b inhibited tumor cell growth, four computational algorithms, namely, DIANAmT, TargetScan, miRWalk and miRanda, were used to determine the potential target genes of miR-216b. Ideal base pairing was observed between the seed sequence of mature miR-216b and 3′-UTR of insulin-like growth factor 2 mRNA-binding protein 2 (IGF2BP2) mRNA; moreover, this seed sequence was highly conserved across species (Figure 3a).

To verify whether IGF2BP2 was a direct target of miR-216b, we subcloned full-length 3′-UTR of IGF2BP2 into a luciferase reporter vector (pMIR-REPORT *β*-galactosidase control vector). By transfecting IGF2BP2 3′-UTR pMIR-REPORT *β*-galactosidase vector into HepG2 cells, we found that miR-216b mimics decreased the IGF2BP2 3′-UTR reporter activity, suggesting that miR-216b specifically targets IGF2BP2. This profound inhibition was abolished when the predicted miR-216b target sequences in the IGF2BP2 3'-UTR were mutated in the Luci-mut vector. Moreover, inhibition of endogenous miR-216b by the addition of the miR-216b inhibitor in SMMC-7721 cells was able to increase luciferase expression (Figure 3b). The changes in luciferase seen all occurred in the absence of any changes seen in the mRNA level of IGF2BP2 (Figure 3c). To directly assess the effect of miR-216b on IGF2BP2 protein expression, we transfected miR-216b into HepG2 cells and found that overexpression of miR-216b reduced IGF2BP2 protein levels. Conversely, miR-216b inhibitor transfection increased IGF2BP2 protein levels in SMMC-7721 cells (Figure 3d).

SMMC-7721 cells were transfected with miR-216b inhibitors. Then, we used RNAi technique to suppress IGF2BP2 expression or used a mock RNA control. CCK-8 assay showed that inhibition of miR-216b in SMMC-7721 cells profoundly decreased their proliferation. In contrast, presence of miR-216b had no significant effect on the proliferation of mock- and vector-transfected SMMC-7721 cells (Figure 3e). These results suggested that IGF2BP2 was necessary for the enhanced proliferation of SMMC-7721 cells induced by miR-216 inhibition.

Next, we performed *in vitro* wound-healing assay to study the motility of the transfected cells. Monolayers of HepG2 cells transfected with miR-216b mimics and HepG2 control cells were scraped using a 2-mm tip, and the gap was photographed at 0, 12, 24 and 48 h by using a confocal microscope. At 6 and 12 h, the gap showed no obvious difference between the two cell groups. At 48 h, cells transfected with miR-216 mimics showed a wider wound than the control cells (Figure 3f). However, the effect of miR-216b mimics was offset in cells transfected with IGF2BP2. These data suggested that the effect of miR-216b on cell motility was dependent on the alteration of IGF2BP2 expression.

We investigated the effect of miR-216b on cell invasion *in vitro*. HepG2 cells transfected with miR-216b alone or together with IGF2BP2 were assessed using Transwell assay. After 48 h, significantly fewer number of miR-216b-expressing HepG2 cells penetrated the lower chamber than control HepG2 cells (*P*<0.05), and this phenomenon was offset by IGF2BP2 overexpression (Figure 3g). In contrast, SMMC-7721 cells transfected with miRNA inhibitors showed significantly higher ability to penetrate the lower chamber than control SMC-7721 cells (Figure 3h).

IGF2BP2 lies upstream of both mitogen-activated protein kinase (MAPK)/extracellular signal-regulated kinase (ERK) and phosphatidylinositol-4,5-bisphosphate 3-kinase (PI3K)/protein kinase B (AKT) signaling pathways through activating insulin-like growth factor 2 (IGF2). To confirm the effect of miR-216b inhibition on IGF2BP2 signaling, we measured the effect of forced expression and inhibition of miR-216b on HepG2 and SMC-7721 cells, respectively. We observed that in HepG2 cells, miR-216b decreased the expression of IGF2 and phosphorylation of AKT, mammalian target of rapamycin (mTOR), and ERK. In contrast, in SMMC-7721 cells, knockdown of miR-216b increased the expression of IGF2BP2 and phosphorylation of AKT, mTOR and ERK (Figure 3i). Further, miR-216b suppressed the proliferation and inhibited viral replication in HepG2.215 cells by targeting IGF2BP2 (Supplementary Figures A, B and C). These data confirmed that miR-216b modified the signaling pathways downstream of IGF2BP2 signaling.

### HBx suppresses p53-mediated activation of miR-216b and upregulates IGF2BP2

**Figure 4:**
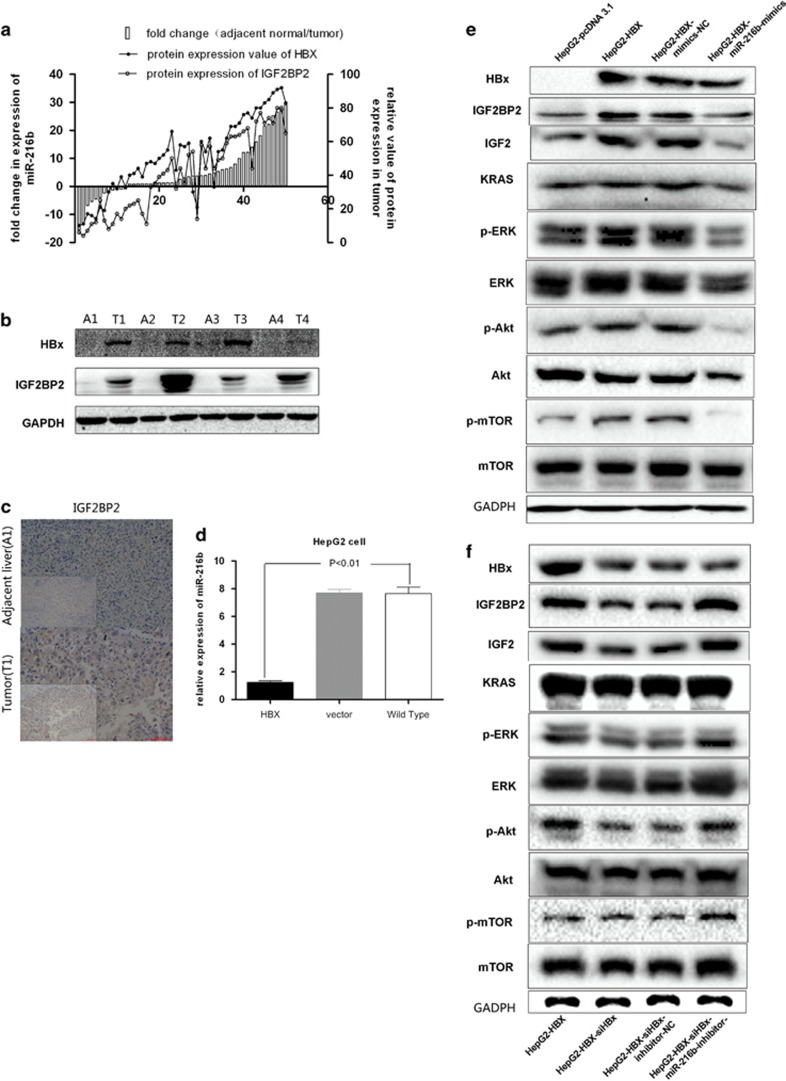
HBx expression correlates with IGF2BP2 expression. (**a**) With the downregulation of miR-216b expression in 50 tumor tissues, the HBx expression and IGF2BP2 expression are increased. (Protein expression was evaluated by IPP6.0). (**b**) The HBx and IGF2BP2 expression are upregulated the same time in HCC tumor tissues than in adjacent liver tissues (A, adjacent liver; T, tumor tissue). (**c**) IGF2BP2 expression is significant higher in HCC tumor tissues with high HBx expression (A1, adjacent liver 1; T1, tumor tissue 1) than in adjacent liver in IHC assay. (**d**) The overexpression of HBx could obviously downregulate the expression of miR-216b in HepG2 cells (*P*<0.01). (**e**) When miR-216b expression is upregulated, the HBx upregulating effects on IGF2BP2 expression and the following pathways are offset. (**f**) When miR-216b expression is downregulated, the siHBx downregulating effects on IGF2BP2 expression and the following pathways are offset

We found that miR-216b expression was strongly associated with HBV infection. HBx has an important role in the pathogenesis of HBV-related HCC. RT-PCR and western blotting showed that expression levels of HBx and IGF2BP2 were upregulated in 50 HCC tissues that showed downregulated miR-216b expression (Figure 4a). Moreover, western blotting and IHC showed that expression levels of HBx and IGF2BP2 in tumor tissues were much higher than those in the adjacent liver tissues (Figures 4b and c). Next, we measured miR-216b levels and IGF2BP2 protein levels in HepG2, HepG2.215 and HepG2-HBx cells (Supplementary Figures D and E). Our results suggested that HBx regulated the expression levels of miR-216b and IGF2BP2. HBx inhibited miR-216b expression in HepG2 cells (Figure 4d), and stimulated IGF2BP2 expression and activated the downstream pathways (Figure 4e). When miR-216b expression was downregulated, siHBx downregulating effects on IGF2BP2 expression and the following pathways are offset (Figure 4f).

**Figure 5:**
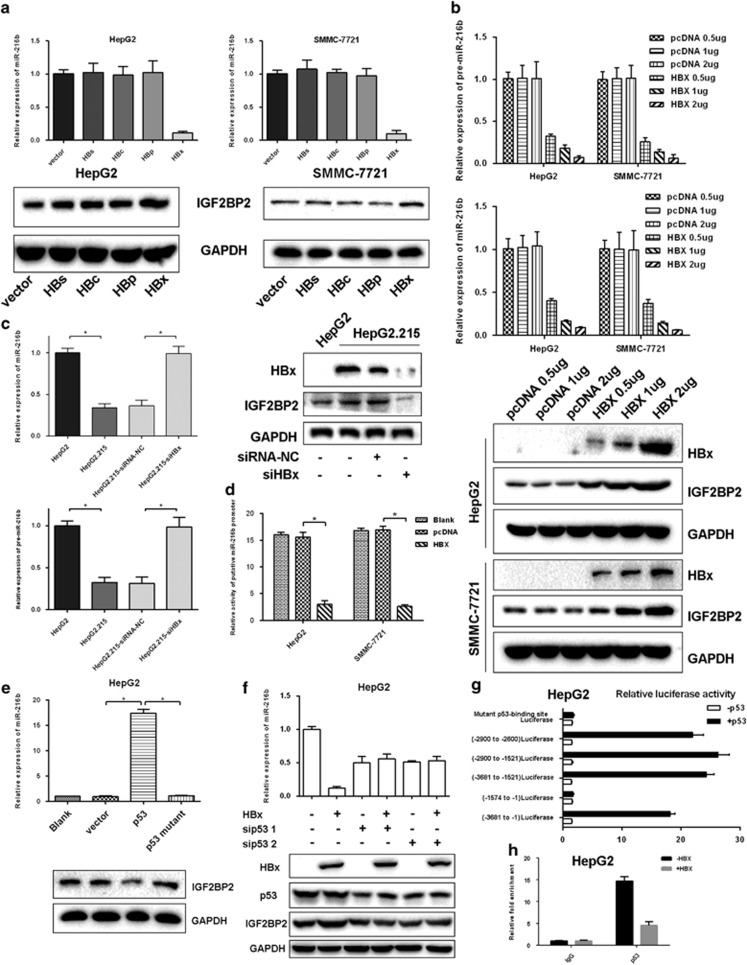
HBx inhibits p53-mediated activation of miR-216b and upregulates IGF2BP2. (**a**) RT-PCR assay for miR-216b and western blotting analysis for IGF2BP2 in vector-, HBs-, HBc-, HBp- or HBx-expressing cells. (**b**) RT-PCR analysis for miR-216b and pre-miR-216b and western blotting analysis for IGF2BP2 in two HCC cell lines, HepG2 and SMMC-7721, transfected with HBx at different doses and controls. (**c**) RT-PCR analysis for miR-216b and pre-miR-216b and western blotting analysis for IGF2BP2 in HepG2 and HepG2.2.15 cells with or without HBx inhibition. (**d**) Dual-luciferase assay of the putative miR-216b promoter in HepG2 and SMMC-7721 cells transfected with HBx and controls. (**d**) HepG2 cells were transfected with p53 or p53 mutant (R249S) and analyzed for miR-216b expression by real-time RT-PCR and for IGF2BP2 expression by western blotting. (**e** and **f**) HepG2 cells were transfected with HBx and p53 siRNAs or control siRNA and analyzed as in **a**. (**g**) ChIP analysis showed that HBx inhibited p53 occupying on the putative miR-216b promoter in HepG2 cells. The data represent the mean±S.D. (*P<0.05)

Next, we examined the effects of four HBV proteins on miR-216b by transfecting HBs, HBc, HBp and HBx in HepG2 and SMMC-7721 cells. At 48 h after transfection, only HBx reduced miR-216b and activated IGF2BP2 expression levels (Figure 5a). Furthermore, transfection of different doses of HBx in HepG2 and SMMC-7721 cells for 48 h reduced the levels of miR-216b and pre-miR-216b in a dose-dependent manner (Figure 5b). Taken together, these results indicated that HBx, but not other HBV proteins, reduced miR-216b in HCC cells. Then siHBx was transfected into HepG2.2.15 cells to silence HBx, which increased the levels of miR-216b and pre-miR-216b (*P*<0.05; Figure 5c). To further investigate the mechanism of miR-216b downregulation by HBx, we examined the effect of HBx on the activity of the putative miR-216b promoter. We found that the activity of the putative miR-216b promoter was reduced in HBx-expressing cells compared with that in control cells (Figure 5d). These results suggested that HBx downregulated miR-216b by regulating its transcription.

HBx regulates gene expression by interacting with host transcriptional factors. Xu *et al.*^14^ used co-immunoprecipitation to confirm the interaction of HBx with p53. Our results showed that overexpression of p53 in HepG2 cells increased the expression of miR-216b and decreased the expression of IGF2BP2. Mutated p53 (R249S) failed to regulate the expression levels of miR-216b and IGF2BP2 (Figure 5e). In contrast, knockdown of p53 decreased the expression of miR-216b and increased the expression of IGF2BP2. Furthermore, knockdown of p53 reduced the ability of HBx to regulate the expression levels of miR-216b and IGF2BP2 (Figure 5f). In addition, p53 stimulated the activity of the luciferase reporter gene containing the putative p53-binding site but did not stimulate the activity of the reporter gene containing a mutated binding site or lacking the putative p53-binding site (Figure 5g). ChIP assay showed that HBx expression decreased the promoter occupancy of p53 (Figure 5h). Taken together, these data strongly suggested that HBx inhibited miR-216b transcription by decreasing the recruitment of p53 to the miR-216b promoter.

## Discussion

MiRNAs were discovered nearly 20 years ago.^15^ Since then, many studies have identified miRNAs showing altered expression levels in neoplastic tissues. MiRNA expression is altered by mechanisms such as deletion, amplification or mutation of miRNA loci; epigenetic silencing; or dysregulation of transcription factors that target specific miRNAs.

Several studies have screened miRNAs in HCC tissues and in the plasma or sera of patients with HCC.^16^ Serum miR-1 and miR-122 are prognostic markers of HCC.^17^ MiR-101 functions as a tumor suppressor by directly targeting Nemo-like kinase in liver cancers.^18^ In this study, we attempted to determine miRNAs that have key roles in HCC occurrence by screening miRNAs in the plasma of patients with HCC who had a family history of HBV-associated HCC. Microarray results identified 26 miRNAs, of which 14 miRNAs, which were not previously reported in HCC, showed >20-fold difference in expression between patients with HCC and healthy volunteers. The remaining 13 miRNAs, which were previously reported in HCC, also showed >20-fold difference in expression between the two groups. Of the identified miRNAs, we focused on miR-216b because its expression showed the highest difference between the two groups and because it was previously identified as a tumor suppressor in other cancers. Forced expression of miR-216b in RInk-1 cells inhibits cell proliferation and colony formation, which is correlated with reduced expression levels of epidermal growth factor receptor and matrix metalloproteinase-14 (MT1-MMP) in pancreatic cancer.^19^ Furthermore, miR-216b is dysregulated in bone marrow mesenchymal stem cells,^20^ and in colorectal cancer cells.^21^ Interestingly, miR-216b is associated with nonalcoholic fatty liver disease.^22^ Despite these findings, the role of miR-216b in HCC has not been reported previously. We found that miR-216b expression was downregulated in HCC tissues compared with adjacent liver tissues and was inversely correlated with tumor size, portal metastasis, HBV infection and prognosis after liver resection.

Cell proliferation assay showed that miR-216b inhibited HCC cell proliferation *in vitro* and *in vivo* by regulating IGF2BP2 expression. IGF2BP2 activated IGF2, which is associated with various effectors, including MAPK and PI3K signaling pathways, and transcription factors such as Ras that are implicated in tumorigenesis.^23, 24^ The importance of IGF2BP2 was highlighted by protein kinase inhibitor sorafenib, which is thought to act by inhibiting KRAS.^25^ IGF2BP2 protein levels were upregulated in HCC cells and tissues and were inversely correlated with miR-216b expression. Luciferase activity assays showed that miR-216b could bind to the 3′-UTR of IGF2BP2 mRNA. We further investigated the effect of miR-216b on IGF2BP2 mRNA levels and found that miR-216b did not modulate IGF2BP2 mRNA level, implying that miR-216b targeted IGF2BP2 by inhibiting its translation and not by degrading its mRNA. Moreover, miR-216b-induced promotion on HCC proliferation was offset by inhibiting IGF2BP2 expression. Further, miR-216b-induced suppression of HCC motility was offset by overexpressing IGF2BP2.

IGF2 is a downstream factor of IGF2BPs, especially IGF2BP2.^26, 27, 28^ Moreover, the MAPK/ERK and PI3K–AKT/mTOR signaling pathways are downstream factors of IGF2.^24^ Our results showed that in HCC cell lines, miR-216b inhibited the activity of the AKT and ERK pathways by targeting IGF2BP2.

MiRNA studies have shown that HBx expression or HBV infection changes the expression of many miRNAs; however, the role of these miRNAs remains largely unknown.^29, 30^ We observed a strong correlation of miR-216b expression with HBV infection and HBx expression levels. HBx overexpression inhibited miR-216b expression and upregulated IGF2BP2 expression. Thus, miR-216b was identified as a downstream target of HBx. Our results showed that HBx regulated miR-216b expression by interacting with the transcription factor p53, suggesting that miR-216b had a role in viral infection. Interestingly, our data showed that miR-216b overexpression inhibited HBV replication and proliferation of HepG2.215 cells (Supplementary Figures A and B). However, the mechanisms through which miR-216b modulates HBV replication remain to be investigated.^31, 32^

In summary, our study suggests that in patients with HCC, miR-216b, which could be transcriptionally reduced by HBx, functions as a tumor suppressor by targeting IGF2BP2 and subsequently suppressing the downstream IGF2, AKT/mTOR and MAPK/ERK signaling pathways. Thus, miR-216b is a fascinating molecule involved in HCC pathogenesis and progression and may serve as a biomarker for early diagnosis of HCC and as a prognostic indicator after liver resection.

## Materials and Methods

### Patients and samples

Blood samples from three patients with HCC who had a family history of HBV-associated HCC were collected at Hepatic Surgery Center, Tongji Hospital, Huazhong University of Science and Technology (Wuhan, China) between September and October 2011. All the three patients were men aged between 35 and 45 years and had at least one sibling who developed HCC and HBV infection in the previous 5 years. Blood samples collected from healthy volunteers without a family history of HCC were used as controls. Fifty sets of HCC tissues and adjacent liver tissues (3 cm away from the tumor margin) were collected from 150 patients with HCC who underwent liver resection at the Hepatic Surgery Center between July 2008 and October 2011. Data on clinical follow-up were collected. Written consent was obtained from all the patients and healthy volunteers before participating in the study, and the study was approved by the Institutional Research Ethics Committee of Tongji Hospital, Tongji Medical College Huazhong University of Science and Technology.

### Microarray and real-time polymerase chain reaction (RT-PCR)

Human miRNA Microarray V16.0 (Agilent Technologies, Santa Clara, CA, USA) was used to identify miRNAs that were differentially expressed between three patients with HBV-associated HCC and one healthy volunteer.

To verify the candidate miRNAs determined using microarrays, plasma miRNAs were obtained from the 10 patients with HCC and 10 healthy volunteers by using mirVana PARIS Kit (Applied Biosystems, Foster City, CA, USA). The identified miRNAs were reverse transcribed using PrimeScript RT-PCR Kit (Takara, Otsu, Shiga, Japan) and were measured using real-time PCR with Realtime PCR Master Mix (TOYOBO, Kita-ku, Osaka, Japan). Expression level of U6 was used as a stable endogenous control. All the assays were performed in triplicate.

Total RNA from HCC tissues and adjacent liver tissues was extracted using TRIzol reagent (Invitrogen, Waltham, MA, USA), and 1 *μ*g of the total RNA was reverse transcribed using the same method as that used for plasma miRNA assay. Expression of candidate miRNAs was determined using StepOne Real-Time PCR System (Applied Biosystems).

### Cell lines, cell culture and transient transfection

L02, SMMC-7721, HepG2, 97-H, LM3, PLC/PRF/5 (PLC) and CT26 were purchased from Typical Training Content Preservation Committee Cell Bank, Chinese Academy of Sciences (Shanghai, China). The cells were cultured in Dulbecco's modified Eagle's medium (DMEM; Gibco BRL, Carlsbad, CA, USA) supplemented with 10% fetal bovine serum and penicillin/streptomycin (100 U/ml) at 37 °C in humidified atmosphere with 5% CO_2_. To promote or suppress miR-216b expression, HepG2 and SMMC-7721 cell lines were transiently transfected using miRNA mimics and inhibitors (RiboBio Co., Ltd, Guangzhou, China). In addition, siRNAs targeting different coding regions of hepatitis B virus x protein (HBx), human IGF2BP2 and p53, and their negative controls (NCs) were purchased from RiboBio Co., Ltd. Transfection was performed using Lipofectamine 2000 (Invitrogen), according to the manufacturer's instructions. Cells were seeded at a density of 5 × 10^4^ cells per well in a six-well plate. Expression of miR-216b was measured using StepOne Real-Time PCR System at 48 h after the cells reached confluence, while that of proteins was measured 72 h after the cells reached confluence.

### Flow cytometry for apoptosis analysis

For apoptosis analysis, 1 × 10^5^ cells per well were seeded in the six-well plates and incubated with DMEM containing 10% serum. After 48 h, floating and adherent cells were harvested and washed twice with pre-cold PBS. The cells were stained for 15 min with Annexin V-fluorescein isothiocyanate and propidium iodide in 500 ul binding buffer and then analyzed by flow cytometry (Becton Dickinson, Franklin Lakes, NJ, USA) within 1 h.

### Luc-UTR vectors, HBx expression vector and IGF2BP2-expressing vector

Full-length 3′-UTR of IGF2BP2 was cloned into *Sac*1 and *Mlu*1 sites of pMIR-REPORT luciferase vector (Ambion, Austin, TX, USA) by using a PCR-generated fragment. Luc-MUT vector, in which the first five nucleotides are complementary to the seed region of miR-216b, was mutated by site-directed mutagenesis (Stratagene, Santa Clara, CA, USA) and was used as a mutant control.

HBx cDNA fragment was amplified from pcDNA3.1-HBx plasmid.^21^ Constructs carrying DNA fragments encoding the other three proteins of HBV (surface antigen (HBsAg), core protein (HBcAg) and DNA polymerase protein (HBp)) were prepared by cloning PCR-derived fragments into pHBV1.3 vector. Full-length IGF2BP2 cDNA that entirely lacked the 3′-UTR was purchased from GeneChem (Shanghai, China) and was subcloned into pcDNA3.1 (+) eukaryotic expression vector (Invitrogen). Empty pcDNA3.1 (+) vector was used as a negative control.

### Western blotting

Proteins were extracted from HCC tissues and cells, and their concentrations were measured. Western blotting was performed using specific primary antibodies against HBx, IGF2, IGF2BP2, ERK, phosphorylated ERK, AKT, phosphorylated AKT, mTOR, phosphorylated mTOR (rabbit-anti human antibody, 1 : 1000; Epitomics, Burlingame, CA, USA), *β*-actin, and GAPDH (1 : 1000; Abcam, Cambridge, MA, USA) and appropriate horseradish peroxidase-conjugated secondary antibodies (1 : 5000; Pierce, Rockford, IL, USA). Protein bands were visualized using an enhanced chemiluminescence detection system (Pierce).

### CCK-8 assay

Transfected and control cells were seeded at a density of 2 × 10^3^ cells per well in a 96-well plate and were incubated at 37 °C for 24 h. Cell proliferation was analyzed at 24, 48, 72, 96 and 120 h by determining the concentration of formazan in cell supernatants with Cell Counting Kit-8 (CCK-8; Dojindo, Kumamoto, Japan), according to the manufacturer's protocols.

### Soft agar colony formation assay

Soft agar colony formation assay was performed using transfected and control cells. Briefly, 1 × 10^3^ cells were equally seeded in a six-well plate containing 0.3% noble agar and were grown for 14 days. Number of colonies was determined by direct counting under an inverted microscope (Nikon, Tokyo, Japan).

### Wound-healing assay and transwell assay

Transfected and control cells were seeded at a density of 5 × 10^5^ cells per well in a six-well plate and were incubated at 37 °C for 24 h. Next, cell monolayers were scraped using a 2-mm tip, and the cells were cultured further in serum-free DMEM. Images were obtained at 0, 6, 12 and 24 h by using a confocal microscope. For the Transwell assay, the cells were trypsinized, counted and resuspended in serum-free DMEM. Approximately 5 × 10^4^ cells were seeded on the upper side of a Transwell chamber (24-well insert, 8- *μ*m pore size; Corning Costar, Lowell, MA, USA) coated with Matrigel. The lower chamber was filled with 10% serum that served as a chemoattractant in DMEM. After 24 h, the cells that had invaded the lower chamber were fixed in methanol, stained with crystal violet and counted.

### Xenograft assay

Twenty 2-week-old male BALB/c nude mice provided by the Experimental Animal Center of Tongji Medical College were divided into four groups, namely, SMMC-7721 control, SMMC-7721 inhibitors, HepG2 control and HepG2 mimics. Exponentially growing HepG2 or SMMC-7721 cells were harvested, and 2 × 10^6^ cells were injected in the bilateral flanks of the nude mice belonging to the four groups. Tumors were formed approximately 14 days after the injection. Mice in the HepG2 mimics and HepG2 control groups received 400 nM of the target RNAs in 0.1 ml every alternate day for 21 days. Mice in the SMMC-7721 inhibitors and SMMC-7721 control groups also received the same concentration and volume of the target RNAs at the same time intervals. Tumors were measured every 3 days by using a vernier caliper. Tumor sizes were calculated on day 21 by using the following formula: average diameter=(longitudinal diameter+transverse diameter)/2.

### Luciferase reporter gene assay

Transcription factor-binding sites in the promoter region of human miR-216b were predicted using JASPAR and ECR browser software. PCR was performed with primers listed in Supplementary Table 1. The putative p53-binding site was 5′-CCATGAAAAGACATG-3′ (from –2691 to –2677 bp), and mutant p53-binding site was 5′-GGTACTTTTCTGTA-3′. For examination of miR-216b promoter activity, serial fragments of human miR-216b promoter region were inserted within pGL3-basic. The plasmids were respectively co-transfected with Renilla luciferase expression vector (pRL-TK) into cells grown in six-well plates. To study the effect of HBx on miR-216b promoter activity, the pcDNA3.1-HBx, pRL-TK and miR-216b promoter-luciferase reporter constructs were co-transfected into cells cultured in six-well plate. All transient transfections were performed using Lipofectamine2000 transfection reagent (Invitrogen) following the manufacturer's instructions. Cells were split and the activities were measured 24 h after transfection using Dual-Luciferase Assay Kit (Promega, Madison, WI, USA).

### Chromatin immunoprecipitation (ChIP) assays

ChIP assay was performed using HepG2 cells transfected with HBx or empty vector by using Magna ChIP Assay Kit (Millipore, Billerica, MA, USA), according to the manufacturer's instructions. Protein–DNA complexes were precipitated using normal IgG and anti-p53 antibodies (Sigma-Aldrich, St. Louis, MO, USA) by overnight incubation at 4 °C in a shaker. Immunoprecipitated DNA was used for real-time PCR amplification.

### Statistical analysis

Differences in survival time, clinicopathological parameters and miR-216b expression between high and low miR-216b expression groups were compared using Kaplan–Meier analysis, log-rank test and *χ*^2^ test. Most clinicopathological parameters for survival were analyzed using univariate and multivariate analyses in Cox proportional hazards regression model. Results of real-time PCR were analyzed using Student's *t*-test. All data were expressed as mean±S.D. *P-*value <0.05 was considered statistically significant. All the experimental data were analyzed using SPSS statistical software (version 16.0; SPSS Inc., Chicago, IL, USA).

## Figures and Tables

**Table 1:** The main plasma miRNAs with obvious differentiation in expression between HCC patients with family history and healthy volunteer based on MiRNABASE16.0 microarray result

*MiRNA name*	*Patient 1*	*Patient 2*	*Patient 3*	*Healthy volunteer*	*Fold change*	*Chr*
hsa-miR-215	4.133392	5.489004	5.329173	−3.01875	277.3476	chr1
hsa-miR-3188	3.99423	3.776789	4.041749	−3.01875	124.5749	chr19
hsa-miR-374a	5.024302	3.531907	−0.24684	−3.01875	121.4428	chrX
hsa-miR-23b	1.58499	3.752035	4.434753	−3.01875	102.9296	chr9
hsa-miR-18a	4.085276	3.931359	1.920744	−3.01875	97.30162	chr13
hsa-miR-4271	1.936976	3.99423	4.005849	−3.01875	96.79688	chr3
hsa-miR-1471	3.859828	2.288063	4.021979	−3.01875	96.30528	chr2
hsa-miR-320a	4.005849	2.026072	3.648332	−3.01875	88.27788	chr8
hsa-miR-188-5p	1.506212	2.23718	4.513003	−3.01875	82.09368	chrX
hsa-miR-1183	3.648332	3.700071	2.258003	−3.01875	81.90769	chr7
hsa-miR-3610	−0.02192	1.58499	4.322987	−3.01875	64.8362	chr8
hsa-miR-199a-3p	2.161674	3.913376	2.009884	−3.01875	63.6739	chr1
hsa-miR-454	4.279344	1.842776	−1.78566	−3.01875	62.93342	chr17
hsa-miR-423-5p	3.752035	2.258003	2.23718	−3.01875	62.05822	chr17
hsa-miR-151-5p	3.931359	2.142199	1.58499	−3.01875	61.2467	chr8
hsa-miR-324-3p	3.776789	1.705998	0.076838	−3.01875	48.69201	chr17
hsa-miR-140-5p	2.310577	2.318683	2.060964	−3.01875	38.15187	chr16
hsa-miR-130b	2.026072	1.952285	2.310577	−3.01875	34.85976	chr22
hsa-miR-197	2.288063	1.910013	1.952285	−3.01875	33.80174	chr1
hsa-miR-148b	2.142199	2.161674	1.795104	−3.01875	33.38865	chr12
hsa-miR-30a	1.60829	1.803955	2.318683	−3.01875	31.14729	chr6
hsa-miR-484	1.892021	1.742625	1.936976	−3.01875	29.41176	chr16
hsa-miR-7	1.705998	1.671912	1.387086	−3.01875	24.48793	chr9
hsa-miR-30c	1.920744	2.088509	−0.02192	−3.01875	24.37926	chr1
hsa-miR-194	0.076838	2.009884	1.653321	−3.01875	22.22771	chr1
hsa-miR-222	1.478218	1.653321	−1.68234	−3.01875	16.86627	chrX
hsa-miR-486-3p	1.300588	1.318576	−1.61597	−3.01875	14.27426	chr8
hsa-miR-221	−0.01614	1.631837	−1.60126	−3.01875	11.93418	chrX
hsa-miR-122	5.884915	8.985012	9.239693	6.08211	5.75829	chr18
hsa-miR-1290	4.739727	5.991765	8.465335	5.489004	3.293882	chr1
hsa-miR-1228	1.910013	4.021979	1.506212	4.677724	0.297525	chr12
hsa-miR-1234	−3.22914	3.488344	−1.54977	3.913376	0.25819	chr8
hsa-miR-18b	2.318683	1.892021	0.042057	3.700071	0.249548	chrX
hsa-miR-126*	2.329368	2.310577	1.631837	4.322987	0.217936	chr9
hsa-miR-191*	−3.22914	1.237284	−1.58659	4.133392	0.053127	chr3
hsa-miR-138-2*	−3.22914	−3.18149	−3.32196	3.488344	0.009411	chr16
hsa-miR-216b	−3.22914	−3.18149	−3.32196	3.752035	0.007839	chr2

**Table 2:** Correlation of miR-216b expression in HCC with 50 patients' clinical and pathological properties

*Parameters*	*Low miR-216b expression (*N*=90)*	*High miR-216b expression (*N*=60)*	*χ*^*2*^*-Test*
Gender	Male (42)-46.7%	Male (24)-40%	*P*=0.861
	Female (48)-53.3%	Female (36)-60%	
Age	≥60 (57)-63.3%	≥60 (24)-40%	*P*=0.183
	<60 (33)-36.7%	<60 (36)-60%	
Tumor size	≥5 cm (42)-46.7%	≥5 cm (9)-15%	*P*=0.044*
	<5 cm (48)-53.3%	<5 cm (51)-85%	
Hepatitis infection	Infected (72)-80%	Infected (30)-50%	*P*=0.026*
	Not infected (18)-20%	Not infected (30)-50%	
HBV DNA quantity differentiation	≥1000 (63)-70%	≥1000 (9)-15%	P=0.001*
	<1000 (27)-30%	<1000 (51)-85%	
	High/moderate (60)-66.7%	High/moderate (48)-80%	*P*=0.479
	Low (30)-33.3%	Low (12)-20%	
Cirrhosis	Present (36)-40%	Present (18)-30%	*P*=0.674
	Absent (54)-60%	Absent (42)-70%	
PVTT	Present (45)-50%	Present (12)-30%	*P=*0.032*
	Absent (45)-50%	Absent (48)-70%	
AFP level>200 ug/l	Yes (33)-36.7%	Yes (15)-25%	*P=*0.578
	No (57)-63.3%	No (45)-75%	
Child–Pugh score	A (81)-90%	A (57)-95%	*P=*0.741
	B (9)-10%	B (3)-5%	

Abbreviations: AFP, alpha-fetoprotein; PVTT, portal vein tumor thrombosis. *Means *P*<0.05

**Table 3:** Univariate and multivariate Cox regression analyses of overall survival of patients with HCC

*Univariate analysis*		*Multivariate analysis*		
*Variables*	P-*value*	*Variables*	*HR (95% CI)*	P-*value*
miR-216b expression	0.015*	miR-216b expression	3.872(0.093–0.995)	<0.049*
Age	0.111			
Gender	0.272			
Tumor volume	0.034*			
HBV infection	0.023*			
Differentiation	0.001*			
Liver cirrhosis	0.124			
HBV DNA quantity	0.015*			
AFPłł level >200ug/l	0.435			
Child–Pugh score	0.713			
PVTT	0.021*	PVTT	7.133 (2.148–145.393)	<0.008*

*Means *P*<0.05

## Abbreviations

HCC: hepatocellular carcinoma
miRNA: microRNA
RT-PCR: real-time polymerase chain reaction
PVTT: portal vein tumor thrombosis
HBV: hepatitis B virus
HBx: hepatitis B virus x protein
HBsAg: hepatitis B virus surface antigen
HBcAg: hepatitis B virus core protein
HBp: hepatitis B virus DNA polymerase protein
IGF2BP2: insulin-like growth factor 2 mRNA-binding protein 2
IGF2: insulin-like growth factor 2
AKT: protein kinase B
ERK: extracellular signal-regulated kinases
mTOR: mammalian target of rapamycin
MAPK: mitogen-activated protein kinase
PI3K: phosphatidylinositol-4,5-bisphosphate 3-kinase
ChIP: chromatin immunoprecipitation

## Notes

We declare all animals received humane care according to the criteria outlined in the "Guide for the Care and Use of Laboratory Animals".

We assume that the corresponding author speaks for all coauthors and certifies that all listed authors participated meaningfully in the study and that they have seen and approved the final manuscript.
