# Supplementary material for: MiR-216b is involved in pathogenesis and progression of hepatocellular carcinoma through HBx-miR-216b-IGF2BP2 signaling pathway
Source: Cell Death Dis. 2015 Mar 5;6(3):e1670–. doi: 10.1038/cddis.2015.46 (PMC4385924; doi:10.1038/cddis.2015.46)
Supplement: Supplementary Table 1 [file cddis201546x1.doc]

Supplement table 1. Primers for different promoter constructs:

| Putative miR-216b promoter  (-3681bp to -1bp) | Sense: 5’- CCCACACAAGCATGGTTAG -3’ |
| --- | --- |
|  | Antisense:5’- CACTTTGAAGTTGTACTTAAGATAG -3’ |
| (-1574bp to -1bp) | Sense: 5’- TCTCATCCTTTGGAGCTTTA -3’ |
|  | Antisense: 5’- CACTTTGAAGTTGTACTTAAGATAG -3’ |
| (-3681bp to -1521bp) | Sense: 5’- CCCACACAAGCATGGTTAG -3’ |
|  | Antisense: 5’- TGCATAGAAAGAAGGGGCTG -3’ |
| (-2900bp to -1500bp) | Sense: 5’- GCAATCACGCTCCTAGGTATTT -3’ |
|  | Antisense: 5’- TGCATAGAAAGAAGGGGCTG -3’ |
| (-2900bp to -2600bp) | Sense: 5’- GCAATCACGCTCCTAGGTATTT -3’ |
|  | Antisense: 5’- ATATTGTTGGAATCATACAGCATTTAG -3’ |

Supplement table 2. The miRNAs has been reported in HCC or others tumors and their differentiation in expression are higher than 20.

| MiRNA name | Fold change |
| --- | --- |
| hsa-miR-215 | 277.34 ↑ |
| hsa-miR-23b | 102.92 ↑ |
| hsa-miR-18a | 97.30 ↑ |
| hsa-miR-199a-3p | 63.67 ↑ |
| hsa-miR-151-5p | 61.24 ↑ |
| hsa-miR-130b | 34.85 ↑ |
| hsa-miR-197 | 33.80 ↑ |
| hsa-miR-148b | 33.38 ↑ |
| hsa-miR-30a | 31.14 ↑ |
| hsa-miR-7 | 24.48 ↑ |
| hsa-miR-30c | 24.38 ↑ |
| hsa-miR-194 | 22.22 ↑ |
| hsa-miR-191* | -18.88 ↓ |

Supplement table 3. The miRNAs has never been reported in HCC and their differentiation in expression are higher than 20.

| MiRNA name | Fold change |
| --- | --- |
| hsa-miR-3188 | 124.5 ↑ |
| hsa-miR-374a | 121.4 ↑ |
| hsa-miR-4271 | 96.79 ↑ |
| hsa-miR-1471 | 96.3 ↑ |
| hsa-miR-320a | 88.27 ↑ |
| hsa-miR-188-5p | 82.1 ↑ |
| hsa-miR-1183 | 81.9 ↑ |
| hsa-miR-3610 | 64.83 ↑ |
| hsa-miR-454 | 62.93 ↑ |
| hsa-miR-324-3p | 48.69 ↑ |
| hsa-miR-140-5p | 38.15 ↑ |
| hsa-miR-484 | 29.41 ↑ |
| hsa-miR-138-2* | -106.25 ↓ |
| **hsa-miR-216b** | **-127.56 ↓** |

Supplement figure. MiR-216b inhibits the viral replication and cell proliferation in HepG2.215 cells. (A) MiR-216b levels and (B) IGF2BP2 protein levels were measured in HepG2, HepG2.215, HepG2-HBx cells. (C) MiR-216b mimics and NC were transfected into HepG2.215 cells, and IGF2BP2 protein levels were measured. (D) RT-PCR analysis for HBV tilter in HepG2.215-NC and HepG2.215-miR-216b cells. (E) CCK-8 assay showed that miR-216b up-regulated HepG2.215 cells have smaller value than control cells.
