# Supplementary figures and images for: MiR-216b is involved in pathogenesis and progression of hepatocellular carcinoma through HBx-miR-216b-IGF2BP2 signaling pathway
Source: Cell Death Dis. 2015 Mar 5;6(3):e1670–. doi: 10.1038/cddis.2015.46 (PMC4385924; doi:10.1038/cddis.2015.46)

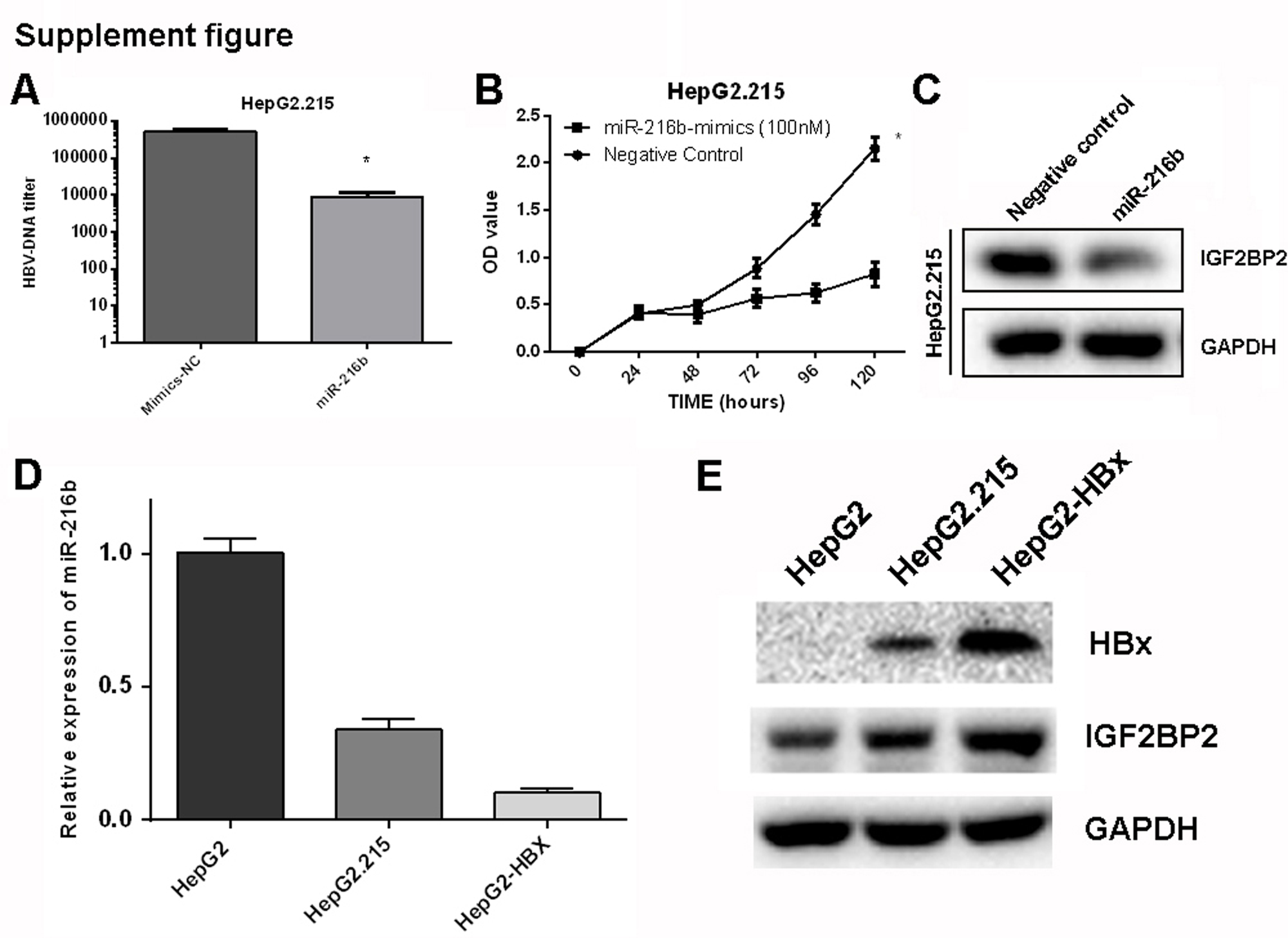

Supplement: Supplementary Figure 1 [file cddis201546x2.tif]
